# Suspected acute coronary syndrome with normal coronary arteries: cardiovascular magnetic resonance in the diagnosis and management in the emergency room

**DOI:** 10.1186/1532-429X-17-S1-O49

**Published:** 2015-02-03

**Authors:** Debora Y Nakamura, Carolina S Reiser, Alejandra Villanueva, Alexandre M Soeiro, Maria Solange A Sanchez, Carlos E Rochitte, Mucio Tavares

**Affiliations:** 1 Cardiovascular Image, Cardiac Magnetic Resonance and, Heart Institute, São Paulo University, Sao Paulo, Brazil

## Background

Acute chest pain, ST-changes on EKG and elevation of cardiac troponin in patients without obstructive coronary artery disease represent a clinical challenge. Cardiovascular magnetic resonance (CMR) can be used to diagnose causes other than obstructive coronary artery disease.The aim of this study was to evaluate the usefulness of CMRto diagnoseconditions in the emergency room that otherwise would be consideredas acute coronary syndrome (ACS) in patients with normal coronary arteries.

## Methods

Forty-seven patients with chest pain and/or electrocardiographic changes and elevated troponin concentration occurring in the absence of significant coronary artery stenosis (normal or stenosis < 50% of the vessel diameter on angiography, computed tomography or both) were selected and prospectively submitted to CMR exam in a 1.5T Philips scanner between May 2013 and June 2014. Ventricular function by cine MR with SSFP technique, and myocardial tissue characterization using late gadolinium enhancement (LGE) were evaluated in patients referred to the Emergency room. LGE patterns were analyzed visually by 2 observers and classified as ischemic (involving subendocardial layer) and non-ischemic (multifocal, not involving subendocardial layer, non coronary distribution).

## Results

Among 47 patients, all with interpretableCMR exams, diagnosis of acute myocarditis was found in 21 patients (45%), acute myocardial infarction in 9 patients (19%) and Takotsubo cardiomyopathy in 4 patients (9%). Other final diagnoses were hypertrophic cardiomyopathy (7%), coronary embolism (4%), cardiomyopathy (4%), sepsis (2%), aortic stenosis (2%) and noncompaction myocardium (2%). In 34 patients (72%), CMR changed theinitial ACS diagnosis to another final diagnosis. Additionally,2 patients (5%) primarily considered as having myocarditis received a final diagnosis of myocardial infarction.

## Conclusions

In the study, 77% of patients had theirprimary diagnosis and treatment changed after CMR study. The presence, distribution and pattern of late gadolinium enhancement by CMR were crucial in establishing a precise final diagnosis and appropriately changing patient management.

## Funding

Not applicable.

